# Molecular docking analysis of zanamavir with haem agglutinin neuraminidase of human para influenza virus type 3

**DOI:** 10.6026/97320630015730

**Published:** 2019-10-31

**Authors:** Chinayan Palani Indumathi, Giridharan Bupesh, Sakthivel Vasanth, Vijayan Senthilkumar, Arumugam Vijaya Anandh, Kanniyan Pandian

**Affiliations:** 1 Department of Virology, King Institute of Preventive Medicine and Research, Guindy, Chennai, India; 2 Research and Development Wing, Sree Balaji Medical College and Hospital, BIHER, Chrompet, Chennai-600044, India; 3 Department of Inorganic Chemistry, University of Madras, Guindy, Chennai-600032, India; 4 R and D Wing, Sree Balaji Medical College and Hospital, BIHER, Chromepet, TamilNadu-600044

**Keywords:** Human para influenza virus, respiratory infections, haem agglutinin, neuraminidase, molecular docking

## Abstract

The human para influenza virus (HPIV) type 3 is an imperative respiratory virus which cause upper and lower respiratory infections. The receptor involved in the viral infection is haem agglutinin neuraminidase. It is of interest to study the interaction of haem agglutinin neuraminidase with zanamavir (4-GU-DANA), a known antiviral drug. We used the PDB structures with PDB IDs 1V2I, 1V3B, 1V3D and 1V3E for studying the interactions with zanamavir. The binding features of zanamavir with 1V2I (1.41kcal/mol) and 1V3E (11.81kcal/mol) having optimal interactions is reported for further consideration.

## Background

Human para influenza virus (HPIVs) type 3 is a member of the respiro virus genus of family Paramyxoviridae, an envelope, non segmented, negative-stranded RNA virus of 15,462 nucleotides [1]. HPIVs are important pathogens, associated with mild upper respiratory tract illness in older children and adults; in infants and young children they are a major cause of morbidity, producing lower respiratory tract illnesses such as croup (inflammation of the larynx and trachea), broncheolitis and pneumonia [2]. Pneumonia accounted for 19% of the 10.6 million yearly deaths in children younger than 5 years in 2000-2003 [3]. HPIVs are not only a common causative agent of (Acute Respiratory Infections) ARI among infants and young children, but these viruses are also associated with nosocomial (hospital borne) acute respiratory illness in the immuno compromised and hematopoietic stem cell transplant patients [4-6]. In USA, it is estimated as 7600 to 48000 among children age, 1 year old and 8100 to 42600 children age 1 to 4 years were hospitalized with HPIVs infection annually [7]. In Southern China it is estimated that HPIVs type 1, 2, 3 and 4 has 2.1% for serotype 3 and other serotypes are less than 2.1% [8]. Among the HPIV, serotype-3 is the most commonly identified HPIV serotype by either viral isolation or antigen detection assay [9,10].

HPIV initiate infection by binding to the surface of the cell receptors through the combined action of two viral surface glycoproteins (haem agglutinin neuraminidase and a fusion protein). These glyco proteins get fused with the surface of the receptors and initiate viral replication process into the host cell cytoplasm and thus more number of virions are produced. During this process, binding to the receptor molecule must trigger the viral fusion protein to mediate fusion and entry of the virus into a cell [11]. The HN protein is present on the cell surface and on the virion as a tetramer composed of disulfide linked dimers [12]. If neuraminidase enzyme is inhibited then that enzyme function is unable to spread the virus to new cells, then the virus infection is obviated and also there is evidence that this enzyme plays a role in the introduction of apoptosis to the infected cells [13-15]. Therefore, it is of interest to study the interaction of haem agglutinin neuraminidase with zanamavir (4-GU-DANA), a known antiviral drug. The binding features of zanamavir and haem agglutinin neuraminidase with optimal interactions is reported for further consideration in this study.

## Methodology

### Receptor data:

The Protein Data Bank (PDB) is a key repository for 3D structure data of large molecules. The 3D data for Hemagglutinin Neuraminidase (HN) of HPIVs type 3 strain in complex with zanamivir (PDB ID is 4MZA and a resolution factor is 1.65Å) by X-ray diffraction method is used in this study. The HPIVs 3 HN protein structure from PDB with PDB IDs 1V2I, 1V3B, 1V3D, 1V3E were used in this study.

### Ligand data:

Zanamivir (4-GU-DANA) is a non polymer type and a modified sialic acid analog with molecular formula is C12 H20 N4 O7. The 3D data for zanamivir from the PUBCHEM database was used in the study.

### Computational methods:

The AUTODOCK software tool was used for docking zanamivir with HN.

### Molecular analysis of HN with 4-GU-DANA:

The five different HN of HPIV type 3 was retrieved from the RCSB Protein Data Bank (PDB). The PDB IDs used were 1V2I, 1V3B, 1V3D, 1V3E AND 4MZA with resolutions 2.2Å, 2.0Å, 2.28Å, 1.89Å and 1.65Å, respectively. The interactions between HN glycoprotein receptors from HPIVs type 3 with 4-GU-DANA was studied using a computational docking method. The binding interactions between 4-GU-DANA and HN glycoprotein were assessed using the AUTO DOCK VINA software.

## Results and Discussion:

**Figure 1:**
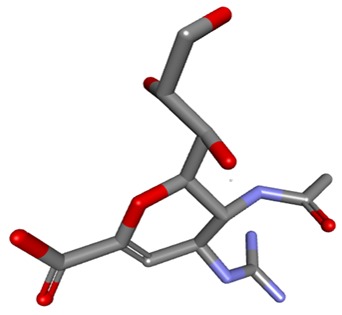
3D structure of Zanamivir (4-GU-DANA).

**Figure 2:**
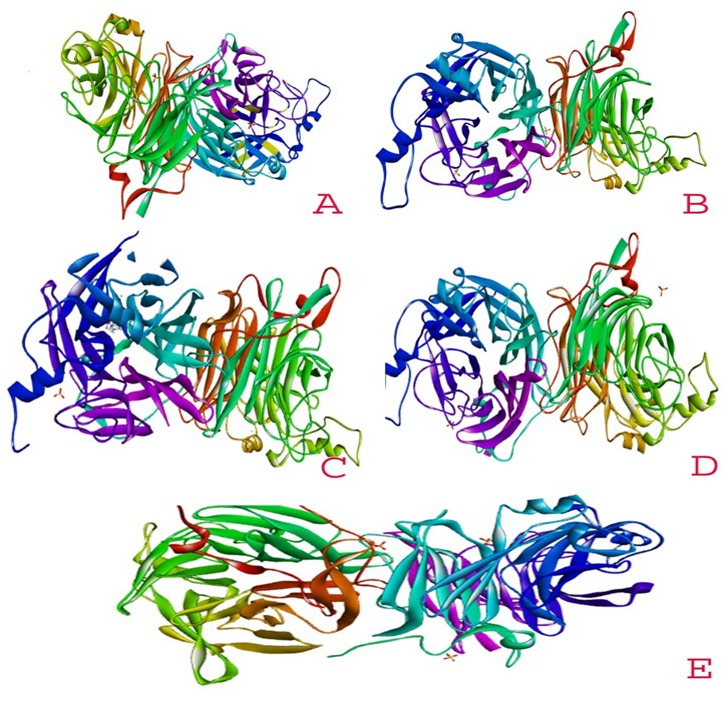
3D Structure of human the para influenza virus (HPIV) type-3 haem agglutinin neuraminidase protein (A) 1V2I, (B) 1V3E, (C) 1V3B, (D) 1V3D, (E) 4MZA).

**Figure 3:**
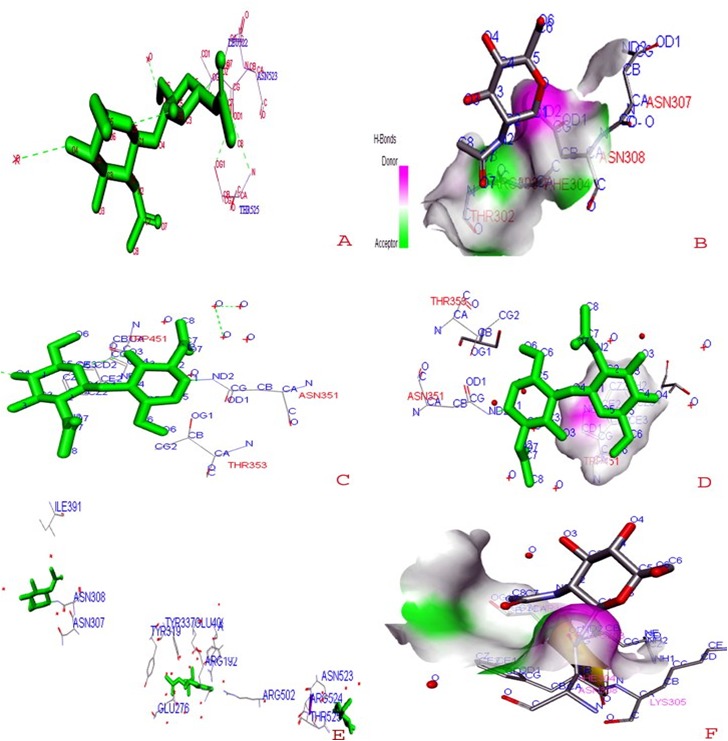
Ligand pocket prediction and molecular docking of HPIV-3 targets (1V2I, 1V3B and 1V3d) with zanamivir ligand

**Figure 4:**
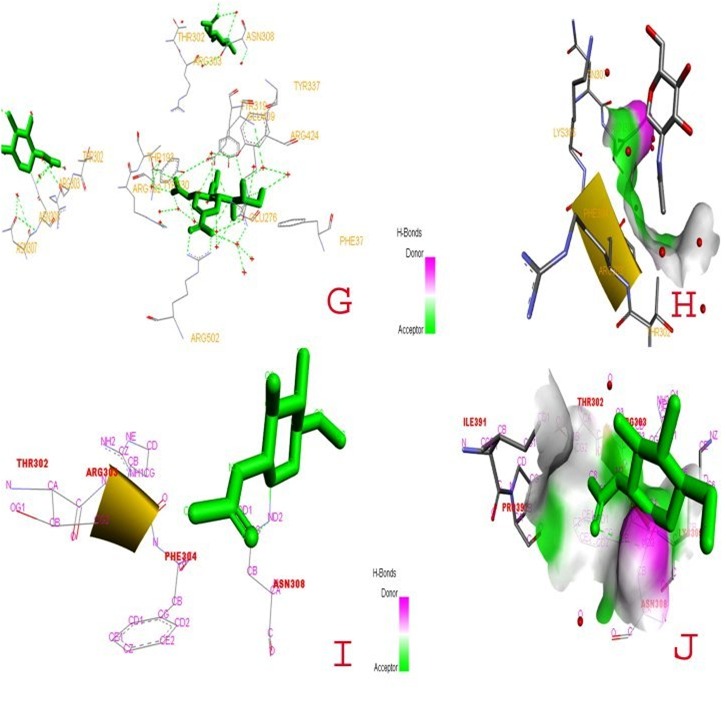
Ligand pocket prediction and molecular docking of HPIV-3 targets (IV3E and 4MZA) with zanamivir ligand

In vitro antiviral activity was evaluated for molecular antiviral activity of zanamvir using HAD inhibition assay, plaque assay and NAI assay (data not shown). However, the mechanism for the antiviral activity remains unknown. Therefore, it is of interest to study the molecular interaction of haem agglutinin neuraminidase with zanamavir (4-GU-DANA), a known antiviral drug. The binding features of zanamavir and haem agglutinin neuraminidase with optimal interactions is reported for further consideration in this study. The ligand structure of zanamivir prepared using the LIGPREP packages is shown in Figure 1. The optimized structures of HPIV- 3 (HN) glycoprotein receptors for molecular docking as shown in Figure 2 are generated using the DISCOVERY STUDIO software. The active binding pockets in the ligand were marked as CA, CB, CG, CE2, CG2 and ND2. Similarly, the active sites of receptors HPIV 3 glycoprotein (PDB ID: 1V2I) are ASN307, 308, THR 302, 304. The active site in 1V3B is made of ASN351, THR 353, 358, TRP 451. The active site in 1V3D is made of ASN307, 308, ILE 391, GLU276, ARG502, 524, TYR19, 337, LYS305 (Figure 3). The active site in 1V3E is made of TYR302, ASN308, ARG303, 307, 308, 424, GLU276, THR193 and GLU276. The active site in 4MZA is made of THR302, ARG303, PHE304, ASN308, PRO392, and LYS309 (Figure 4).

The molecular docking features of HN glyco proteins with zanamivir are given in(Table 1). Results show that 1V2I docked well with 96.6% frequency and 1.41 Kcal/mol of total inter molecular energy having an inhibition constant of 496.10 µM. 1V2I showed the highest fit with a docking score of 11.81kcal/mol. The next best docking in 1V3E having 84.95 docking score,-2.88 total inter molecular energy, electrostatic energy (-0.87) and the free energy binding as 138.78 Kcal/mol is reported. 1V3B shows good docking with zanamivir having 81.77% docking frequency and total inter molecular energy is -2.94 kcal/mol, where electrostatic energy is -0.8 Kcal/mol and the free energy binding is 145.3 Kcal/mol. 1V3D docked with 77.13% frequency having -0.98 total inter molecular energy where -1.47 Kcal/mol is electrostatic energy and the free energy binding is 103.43 Kcal/mol. 4MZA docked with a frequency of only 58.89%. The total intermolecular energy is 1.81 Kcal/mol where electrostatic energy is -0.92 Kcal/mol and the free energy binding is 60.58kcal/mol. High throughput screening (HTS) is applied for the effective drugs to inhibit the activity of viral receptors and nearly 32000 compounds were identified as potent drugs against several viral receptors [16-19]. The molecular docking analysis data of zanamivir with the HN target protein provides sufficient insights for the design and development of an effective lead molecule against HPIV.

## Conclusion

We report the binding features of zanamavir with PDB ID: 1V2I (1.41kcal/mol) and PDB ID: 1V3E (11.81kcal/mol) having optimal molecular interactions for further consideration towards the design and development of an effective lead molecule against HPIV.

## Figures and Tables

**Table 1:** Molecular interaction features of zanamivir with HN glycoprotein

Rank	Est.free energy of binding (kcal/mol)	Est.inhibition constant (Ki)	Vdw + Hbond + desolv energy (kcal/mol)	Electro Static Energy (kcal/mol)	Total Intermolecular energy (kcal/mol)	Frequency	Interact surface
1v2I	11.81	496.10µM	0.5	-2.03	1.41	96.60%	-58.71
1v3e	138.78	456.0 µM	-0.65	-0.87	-2.88	84.95%	-219.74
1v3b	145.03	458.90 µM	-0.68	-0.8	-2.94	81.77%	-229.16
1v3d	103.43	514.20 µM	-0.31	-1.47	-0.98	77.13%	-30.67
4mza	60.58	496.20 µM	-0.43	-0.92	1.81	58.89%	-134.63
